# Impairment of Proprioception in Young Adult Nonradicular Patients with Lumbar Derangement Syndrome

**DOI:** 10.1155/2021/5550257

**Published:** 2021-10-07

**Authors:** Marzena Olszewska-Karaban, Anna Permoda-Białozorczyk, Aneta Dąbrowska, Ewa Bandurska, Andrzej Permoda, Jolanta Zajt, Agnieszka Sobierajska-Rek, Dominika Szalewska

**Affiliations:** ^1^Clinic of Rehabilitation Medicine, Faculty of Health Sciences, Medical University of Gdańsk, Gdańsk, Poland; ^2^Biomed Rehabilitation Center, Dębinki 7d, 80-294 Gdańsk, Poland; ^3^Gdańsk College of Health, Pelplińska 7, 80-335 Gdańsk, Poland; ^4^Acting Head of Center for Competence Development, Integrated Care and e-Health, Medical University of Gdańsk, Dębowa 30, 80-208 Gdańsk, Poland; ^5^Gdańsk University of Physical Education and Sport, Kazimierza Górskiego 1, 80-336 Gdańsk, Poland

## Abstract

Maintaining body balance is a complex function based on the information deriving from the vestibular, visual, and proprioceptive systems. The aim of the study was to evaluate quiet single stance stability in young adults with lumbar derangement syndrome (LDS) and in the control group of the healthy subjects. The second aim of this study was to determine whether pain intensity, degree of disability, and the level of physical activity can influence postural control in patients with LDS. It is important to underline that selecting a homogeneous group of LBP patients using, for example, mechanical diagnosis and therapy method and Quebec Task Force Classification, can result in an increased sensitivity of the study. The study included 126 subjects: 70 patients with LDS (37 women, 33 men) and the control group 56 healthy volunteers (36 women, 20 men). In case of multiple group comparisons for variables with normal distribution, ANOVA post hoc test was used or, as the nonparametric equivalent, Kruskal-Wallis test. In all these calculations, the statistical significance level was set to *p* < 0.05. The stability index eyes open for the study group was 88.34 and for the control group 89.86. There was no significant difference in the level of postural control between the study and control groups (*p* > 0.05). The level of stability index eyes closed (SI EC) for the study group was 71.44 and for the control group 77.1. SI EC results showed significant differences in proprioceptive control during single leg stance between the study and control groups (*p* < 0.05). The level of pain intensity, the degree of disability, and physical activity level did not influence postural control in the study group with LDS. In summary, patients with LDS showed significantly worse proprioceptive control.

## 1. Introduction

Postural control is the ability of regaining body balance in space, lost as a result of destabilising stimuli, such as physical activity or external forces, while the interaction with the surroundings. Postural control is also defined as the ability to maintain standing position and to stabilize the centre of gravity of the human body within the base of support [1, 2]. Maintaining postural stability is a complex function involving a variety of neuromuscular processes, which are dependent on sensory input from the vestibular, visual, and proprioceptive systems [3]. Proprioception is a key component of the somatosensory system which, with the speed of 80-120 m/s, by means of muscles, tendons, joints, and fascia receptors, transfers information about body position to the central nervous system (CNS) [4]. Proprioception is crucial in the stability of joints and prevention of injury [5, 6]. The decreased proprioceptive control in patients with LBP influences postural strategy and can cause balance disorders [7, 8], which may be the most serious consequence of LBP [8, 9]. In addition, according to Pedersen et al., proprioceptive feedback from muscle spindle afferents is negatively affected by nociceptive afference [10] which may also contribute to increased postural inclination.

Many scientific studies on postural control in LBP patients have been conducted so far. The results of these studies are inconclusive [9, 11]. The inconsistencies may be caused by shortcomings in standardization of balance control evaluation in patients with LBP. The discrepancies may also be caused by various sensory conditions and difficulty of experimental conditions.

As far as vision is concerned, in comparison with patients suffering from NSLBP (eyes open conditions), centre of pressure (COP) excursions increased under visual deprivation, which supports the previously mentioned proprioceptive deficits in NSLBP patients [9]. Eyes closed conditions obviously challenge an existing impaired sensory input from muscles and joints. Low-frequency disturbances [12], occurring while patients perform a quiet stance, are primary controlled by vision.

Test results may be inconsistent also due to sample size, age, or BMI. It is known that the physical parameters of the body (size, mass distribution, and inertial properties of the body segments) can partly explain the behavior of postural sway. Postural control may be dependent on the centre of mass orientation resulting in worse balance in taller subjects [13].

Some research reports that older patients with LBP present greater sway of the trunk in comparison with healthy controls at the same age [14].

Moreover, postural control is poorer in older adults with LBP than in younger adults with the same problem [15].

There are also findings that obese patients have poorer postural control than underweight, normal weight, and overweight groups during the bilateral and unilateral stance tests [16].

On the other hand, many of the studies finding increased sway did not adequately account for potential confounders such as age and body height and mass.

Thus, the main aim of our study was to compare postural control in young healthy adults and in young patients with lumbar derangement syndrome (LDS) during quiet standing.

The level of physical activity, disability, and different levels of pain intensity in LBP patients may negatively affect the level of postural control.

Studies find a correlation between higher severity of LBP and the increased sway; however, fear of the increase of LBP during balance test may cause the improvement of postural control as a result of higher level of muscles cocontraction [17].

Therefore, the second aim of this study was to determine whether pain intensity, degree of disability, and the level of physical activity can influence postural control in patients with LDS.

LBP is still the main cause of chronic pain and disability.

There are numerous causes of LBP and, therefore, a variety of symptoms and pain topography. The area of pain, causes of LBP, and duration of symptoms can also affect the level of neuromuscular control. Thus, heterogeneity in LBP patients can also affect the results of examination of body balance.

As stated in the systematic review of Mazaheri et al., the sample size had a tendency to be larger in studies not showing an effect, which may indicate that stricter inclusion leads to either more homogeneous groups or more strict experimental control in the smaller studies leading to an increased sensitivity [11].

Hence, we wanted to underline that selecting a homogeneous group of LBP patients using the mechanical diagnosis and therapy method (MDT) [18] and Quebec Task Force Classification (QTFC) can lead to a higher sensitivity of the study [19, 20]. In conclusion of this above, we believe that this article will highlight the causes of discrepancy in the results of the body balance tests and underline the necessity of applying a reliable examination in inclusion criteria of LBP patients. We also believe that the assessment of postural control in patients with recurrent LBP is still necessary to eliminate the risk of new low back pain episodes, which may initially occur as incidental episodes leading to permanent disability.

## 2. Materials and Methods

### 2.1. Participants

Subjects from the study group were patients with LDS, treated in the Neurosurgery Clinic at the University Clinical Centre in Gdansk. Subjects from the control group were the healthy volunteers, who responded to the invitation for the examination. The participants of the study and control groups met the inclusion criteria (see Table 1). The minimum sample was determined on the basis of the pilot examination of 25 patients with LDS, to define the minimum sample size in order to obtain 80% of the power analysis. These results of postural control were compared to the standards defined by the producer of the device.

People with pain lasting at least 12 weeks or longer were enrolled in the study. So these were patients with chronic LDS. Patients with BMI greater than or equal to 30 were excluded from the study because excess body weight may have a significant impact on balance control; the disturbance of which increases the risk of LBP [21]. On the other hand, an increased body mass index is a significant risk factor for low back pain [22].

The study was approved by Bioethics Committee of the Medical University in Gdańsk. All volunteers (the control group) and patients provided written informed consent in accordance with the procedures approved by the agreement of Bioethics Committee of Medical University of Gdańsk.

### 2.2. Methods

#### 2.2.1. Qualifying Patients with LDS to the Study Using MDT and QTFC

**Figure 1:**
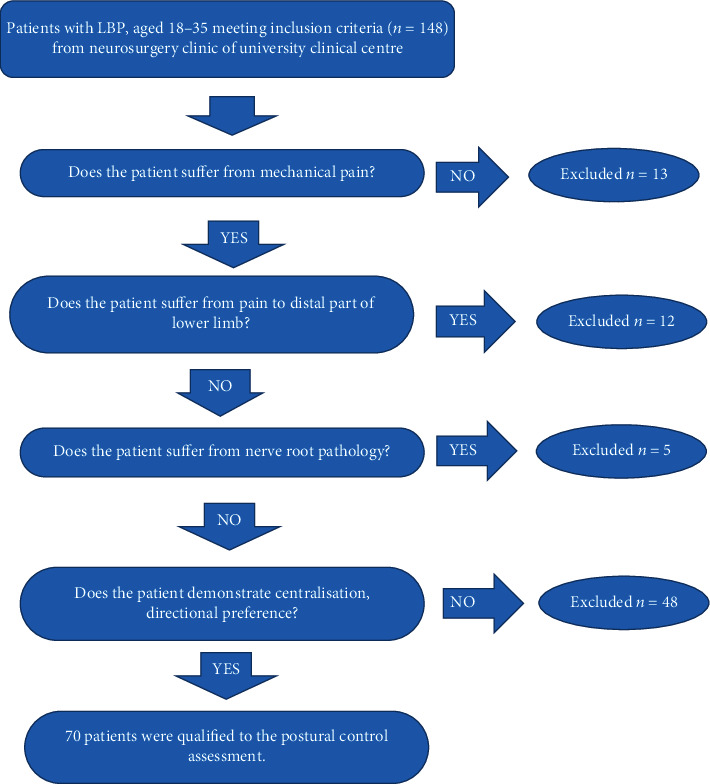
Study flowchart in the study group on the basis of MDT and QTFC.

After interviewing the subjects for the inclusion and exclusion criteria, the study group was examined with the use of the procedure according to the Mechanical Diagnosis and Therapy (MDT). The examination was conducted by a certified therapist of the method. The study group included subjects with directional preference and centralisation of symptoms [18]. MDT method, taking into account clinical pain syndrome, enables reaching diagnostic validity and reliability with the high level of consistency between therapists examining the same patient [23–25]. In the directional preference, one direction of movement increases pain, and the movement performed in the opposite direction reduces the pain. Centralisation of symptoms occurs in repetitive movements, when the symptoms are gradually eliminated in the proximal direction, to the complete relieving. Directional preference and centralisation of symptoms are a scientifically proven phenomenon [26, 27]. Due to MDT classification, only patients with mechanical pain, directional preference, and centralisation of symptoms were included in the study, excluding subjects with pain related to an inflammatory process. In connection with the above, only patients with lumbar derangement syndrome (LDS) were qualified for the further examination of postural control on Delos Postural Proprioceptive System (DPPS). The study group was also standardized in terms of topography of pain with the use of the Quebec Task Force Classification (QTFC) [19, 20]. Patients with low back pain without radiation—41 subjects (58%: number 1 category according to QTFC) and with radiation to the proximal part of lower limb (to the level of knee joint) and 29 subjects (42%: number 2 category according to QTFC)—were qualified for the further examination. Patients with the symptoms to the distal part of the limb, sciatica, and neurological deficits were excluded from the further examination of postural control.  Figure 1 shows the scheme of inclusion of patients with LDS in the examination.

#### 2.2.2. Postural Stability Assessment

**Figure 2:**
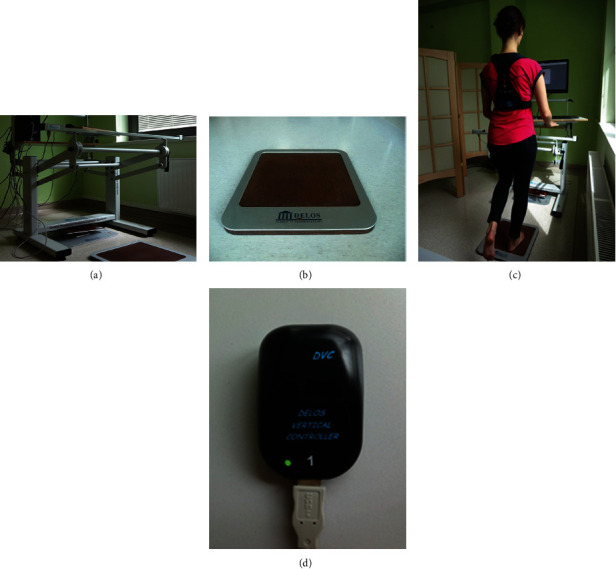
Delos postural proprioceptive system: (a) the postural proprioceptive station, (b) Delos Equilibrium Board, (c) position during Static Riva Test, and (d) Delos Vertical Controller.

Postural stability was assessed with the use of the device Delos Postural Proprioceptive System (DPPS software version 6.5, Delos, Turin, Italy) (see Figure 2(a)). DPPS also enabled the assessment of the fall risk and conducting a proprioceptive training. To assess postural control, Static Riva Test (SRT) was used. The examined subject was standing barefoot on a stable, wooden surface of a Delos Equilibrium Board (DEB) (see Figure 2(b)) with the knee of the supporting leg bent to 10°, while the untested leg remained in 45° knee fixation (see Figure 2(c)). No feedback on postural stability was given during the test. SRT comprised six trails—two with eyes open (first on the left leg (LL) and then on the right leg (RL)) and four trials with eyes closed (LL, RL, LL, and RL). The electronic postural reader Delos Vertical Controller (DVC) was placed on the subject's sternum (see Figure 2(d)). DVC is a two dimensional sensor of the acceleration, measuring the degree of an average inclination of the trunk in the frontal (*x*) and sagittal (*y*) planes (Pi*xy*). When it was necessary (to avoid the fall), the examined patient could support themselves over a metal bar, touching it for as short period of time as possible. The metal bar of Delos Postural Assistant (DPA) is equipped with an infrared sensor recording the time and the number of hand supports. The information derived from DVC and DPA is processed and analysed in real time by the postural system analyzer (PSA), which is special DPPS software. Prior to the test, the patient was familiarised with the test procedure and performed the propaedeutic test. In the examination the subject was instructed to stand as still as possible. Each trial lasted 20 seconds with a 15-second break after each trial. All tests were conducted in the same lighting conditions, in a quiet and undisturbed room.

#### 2.2.3. The Postural Control Ratio

The postural control ratio is the postural stability level achieved during trials with the eyes open. The stability index eyes open (SI EO) is an average of the results achieved during single leg stance on the left and then on the right leg. The stability index (SI) was determined on the basis of trunk inclinations ranging from 0 to 100%. The autonomy of system is the time when there is no subject's hand contact with DPA. Patients with well-functioning postural control demonstrate stability of body position in both, static and dynamic loss of balance, keeping the head and trunk in an almost stable position. It is proved by the low ratio of an average postural instability (Pi*xy*) and the complete autonomy of the system reflected by no DPA use for the loss of body balance. The algorithms for calculating the above values are available in the article by Riva et al. [28].

#### 2.2.4. The Proprioceptive Control Ratio

The proprioceptive control ratio is the postural stability level achieved during trials with eyes closed. The stability index eyes closed (SI EC) is an average of best results achieved during single leg stance on the left and then on the right leg. Averaging results eliminated domination of one limb, as well as, i.e., the influence of unilateral low back pain and/or lower limb, or injuries that occurred in the past. A high rate of the proprioceptive control ratio is typical for the correct postural control, maintained during single leg stance without visual control, although simultaneous activation of the vestibular system cannot be excluded. The loss of balance occurs more frequently during trials with eyes closed; accordingly, the time of support for regaining stability is longer and the rate of inclination of the trunk (Pi*xy*) higher.

#### 2.2.5. The Statistical Analysis

All calculations were carried out by means of Microsoft Excel spreadsheet ver.2007 and STATISTICA, StatSoft, Inc. ver.8.0 statistical package (data analysis software system). In the statistical description of quantitative data classical measures of location, such as arithmetic means, median, and mode along with the measures of variation, such as standard deviation and range, were used. The normality of the distribution of variables and variance equality of a studied feature in groups was tested by the use of Shapiro-Wilk's test and variance equality test. In order to compare groups in pairs for quantitative and discrete data, Mann–Whitney *U* test was used. In case of multiple group comparisons for nonparametric data, the Kruskal-Wallis test was used along with Dunn's test as post hoc test, as the nonparametric equivalent of ANOVA. In all statistical tests the statistical significance level was set to *p* < 0.05.

## 3. Results

The examination of postural control was conducted in 126 subjects. The study group consisted of 70 patients with LDS (37 women, 33 men) aged 18-35 (the average age: 28.35; SD 4.19), and the control group consisted of 56 healthy volunteers (36 women, 20 men) at the age of 18-35 (average age: 25.16; SD 4.34).  Table 2 shows the characteristic of the study and control groups.

The characteristics of the study and control groups as regards the degree of disability (Oswestry Disability Index (ODI), pain (Visual Analogue Scale (VAS)), and physical activity level (International Physical Activity Questionnaire (IPAQ)–long version) are presented in Tables 3–5, respectively.

### 3.1. Postural and Proprioceptive Control

**Figure 3:**
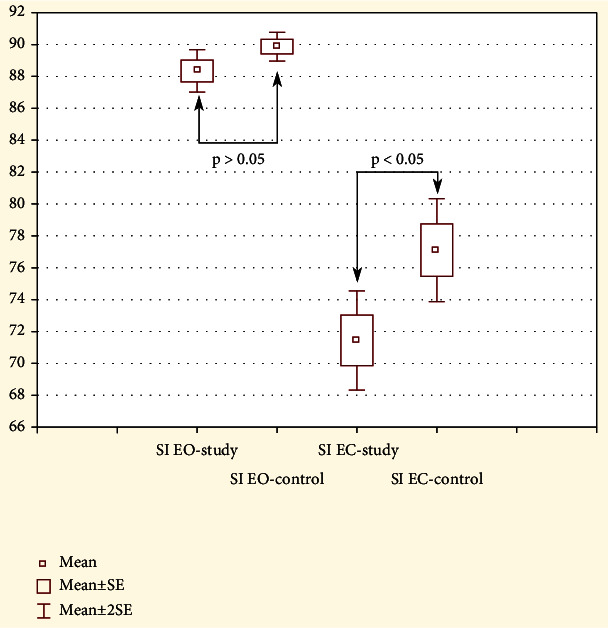
Graphical comparison of the mean stability indexes between the study group and the control group. Static Riva Test: stability index in the study group and control group. SI EO: stability index during single leg stance with open eyes—postural control ratio; SI EC: stability index during single leg stance with closed eyes—proprioceptive control ratio.

Static Riva Test (SRT) enabled the assessment of postural stability in the study group with lumbar derangement syndrome (LDS) and in the control group during single leg stance. SI EO for the study group was 88.34 (SD 5.69) and for the control group 89.86 (SD 3.43). There was no significant difference in the level of postural control between the study and the control group, *p* > 0.05 (see Figure 2). The level of SI EC for the study group was 71.44 (SD 13.26) and for the control group 77.1 (SD 12.32). SI EC results showed significant differences in proprioceptive control during single leg stance between the study and the control group, *p* < 0.05 (see Figure 3).

### 3.2. The Impact of Pain

**Figure 4:**
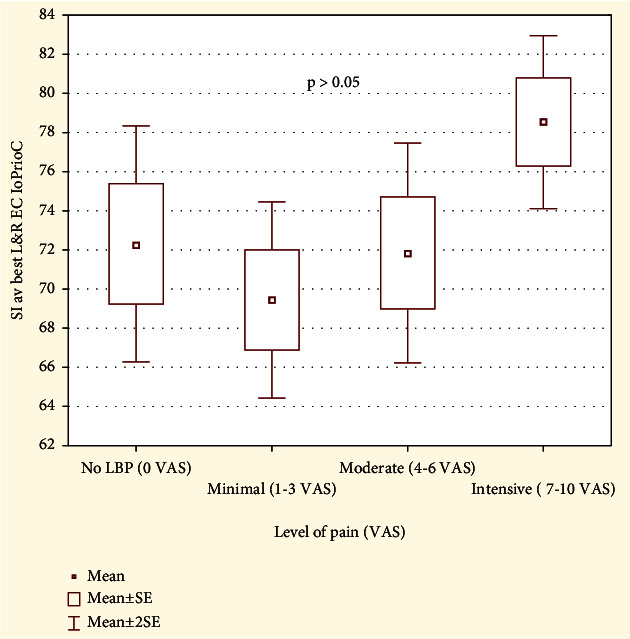
The comparison of the proprioceptive control ratio in the study group depending on pain intensity.

We assessed the influence of pain intensity (VAS) on the level of stability index eyes closed in the study group. In the group with no pain, the level of SI EC was 72.31 (SD 15.37), in the group with minimal pain 69.44 (SD 12.81), in the group with moderate pain 71.84 (SD 11.8), and in the group suffering from intense pain 78.53 (SD 3.9). Kruskal-Wallis test did not show statistically significant differences of the SI EC depending on pain intensity in the study group, *p* > 0.05 (see Figure 4).

### 3.3. The Impact of Disability

**Figure 5:**
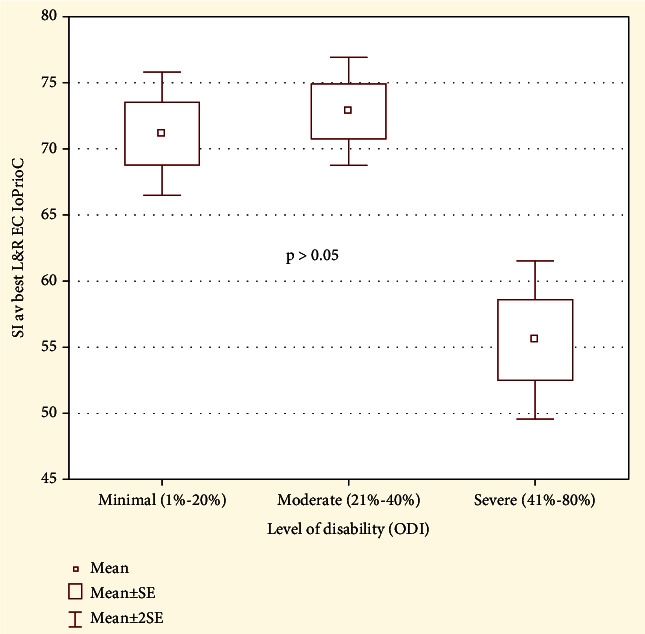
The comparison of proprioceptive control ratio in the study group depending on the degree of disability.

We assessed the influence of disability degree (ODI) on the stability index eyes closed (SI EC) in the study group. In the group with minimal disability, the level of SI EC was 71.13 (SD 14.45), in the group with moderate disability 72.89 (SD 11.6), and in the group with severe disability 55.55 (SD 4.3). Kruskal-Wallis test did not show statistically significant differences in the level of SI EC depending on the disability level in the study group, *p* > 0.05 (see Figure 5).

### 3.4. The Impact of Physical Activity

**Figure 6:**
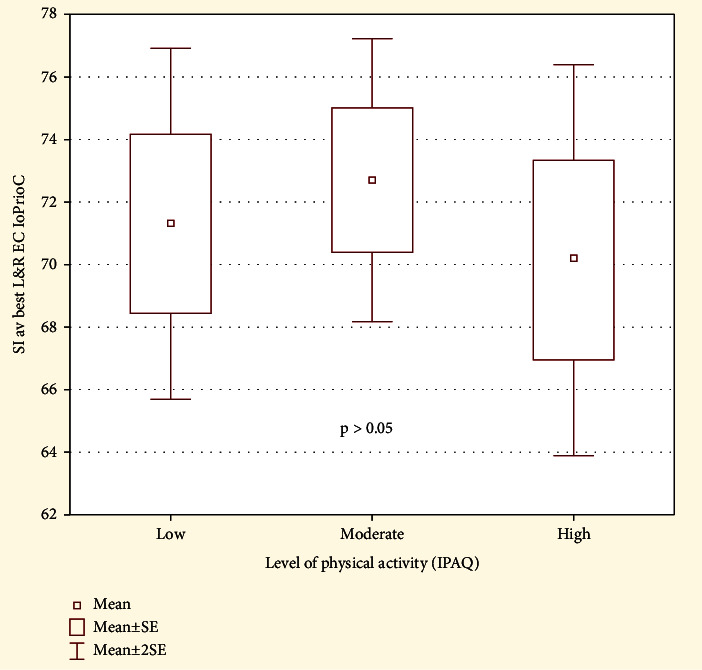
The comparison of the proprioceptive control ratio in the study group depending on the level of physical activity.

We assessed the influence of physical activity level (IPAQ) on the stability index eyes closed in the study group. In the group with low physical activity, the level of SI EC was 73.3 (SD 14.12), in the group with moderate physical activity 74.99 (SD 12.23), and in the group with high physical activity 73.53 (SD 13.21). Kruskal-Wallis test did not show statistically significant differences in the level of SI EC depending on the level of physical activity in the study group, *p* > 0.05 (see Figure 6).

## 4. Discussion

The main aim of our study was to assess postural control in patients with lumbar derangement syndrome (LDS). The level of stability index eyes open (SI EO) during single leg stance is postural control ratio. The results of SI EO did not indicate the deterioration of postural control in patients with LDS in comparison with the control group. The level of stability index eyes closed (SI EC) during single leg stance is the proprioceptive control ratio. The results of SI EC indicated the deterioration in proprioceptive sense in patients with LDS, in comparison with the control group. The results of stability index assessment in the group with LDS during trials with eyes open and closed clearly indicate the enhanced role of the visual system in the process of maintaining body balance and stability. In this case, movement without visual control leads to performing excessive range of inclination.

Current reports regarding postural control in patients with LBP are inconclusive [9, 11]. Our findings are partially confirmed by the results of the research by Sell et al. indicating that patients with a history of LBP show significantly worse balance control with eyes open and closed [21]. On the other hand, the study of Jo et al. revealed statistically significant differences between the LBP group and the control group during single leg stance with eyes closed [29]. Another research suggests that postural stability with eyes open might be impaired only in patients with severe LBP [30]. The fact that there is no difference in postural control between the study and the control group in our research may be caused by low prevalence (4%) of patients with severe pain. However, in Tsai's research there were no significant differences between the LBP group and the control group while single leg stance with eyes open and closed [31]. The inconsistency of abovementioned studies may be explained by the hypothesis of existence of subgroups presenting differences in muscular activity and movement patterns in the population of subjects with LBP. It is observed that some of LBP patients may present increased cocontraction and increased reflex gains with small centre of pressure (COP) displacements. On the other hand, there are patients that exhibit a decrease of muscular activity and large COP displacements. These subgroups are a part of the continuum, and between them and patients with LBP, the impairment of postural control may be absent [32].

The second aim of this study was to determine whether pain intensity, the degree of disability, and the level of physical activity can influence postural control in patients with LDS. Our study did not show statistically significant differences between the intensity of pain (VAS) and the level of SI EC in the group with LDS. Most likely, it was due to a slight intense of pain during balance control examination as a result of using the MDT method for diagnostic purposes. There is an evidence that patients with high intensity of pain (7-8 points in 11-point numerical rating scale) are characterised with higher COP inclination [33]. At lower and medium pain intensities, there is no significant change in the COP parameters [33].

In our study, no statistically significant differences were also found between the level of physical activity (IPAQ long form) and the level of the SI EC in the group of patients with LDS. Moreover, we did not reveal worse postural control in patients with low physical activity level. In contrast to our results, Alsufiany et al. reported the relationship between physical activity (IPAQ short form) and static control during left single stance with eyes closed in patients with nonspecific chronic low back pain [34]. However, they neither revealed correlations with static postural control with eyes open nor with dynamic postural control [34].

We did not observe statistically significant differences between the degree of disability (ODI) and the level of the SI EC in the group of patients with LDS. Similarly, the study of Brech et al. showed no correlations between the intensity and the frequency of pain and the degree of disability for the balance measurements in women with chronic low back pain [35]. However, it revealed significant correlation between the degree of disability (ODI) and the inclination speed on a stable surface with eyes open [35].

In our study, despite a low level of pain and a low degree of disability, patients with LDS in comparison with healthy subjects showed significant differences in postural control during single leg stance without visual control. The results may indicate that worse postural control in patients with LDS is not caused by pain, disability, or physical activity level, but it reflects the development of alternative strategies for body postural management. Results from other studies suggest that patients with LBP present more ankle-steered proprioception with less upweighting lumbar proprioception [36, 37]. These alterations may lead to the chronification of LBP [38]. Several neuroimaging studies revealed correlations between the motor control impairment and structural brain changes such as reduced microstructural integrity of the superior cerebellar peduncle or cortical thinning of the anterior cingulate cortex in patients with chronic LBP [39, 40].

In our article, we would like to draw attention to the methodology of postural control assessment in patients with LBP, as numerous studies concerning the assessment of postural control in patients with LBP are inconclusive. The possible cause of the inconsistencies is the differences in methodology of the examination as well as the multiplicity of measurement devices and tests. Systematic review by Mazaheri et al. does not provide clear response on postural control in patients with LBP in comparison with the healthy subjects [11]. In 2019, Koch and Hansel, the authors of a systematic review, underlined the importance of standardization of diagnosis procedure, which would enable to draw more reliable conclusions from the examination of postural control in patients with LBP [41].

### 4.1. Then, How Can More Reliable Conclusions from the Examination of Body Posture Control with LBP Be Drawn?

Like Preuss et al., we believe that spine functions should not be considered separately from the lower limbs; therefore, standing position seems to be the best condition for body posture stability measurement [42]. The examination of proprioceptive lumbar spine control should include trials in static and/or dynamic conditions in single leg stance, since maintaining balance in this position is an additional difficulty for the patient in comparison to more stable position of double leg stance. Luoto et al. underline that the strong link between LBP and postural control was discovered while single leg stance, when the loss of postural control is more likely to occur [30]. Body stability while single leg stance is important as during a march at a normal speed single leg stance covers 80% of the walking cycle [28].

Maximal postural control while single leg stance guarantees security while walking, jogging, jumping, and performing more complicated motor tasks. The stability during single leg stance should be based mainly on proprioceptive control (with minimal engagement of visual and vestibular systems). When visual control is excluded, it cannot compensate weakened proprioceptive control. Thus, while examining postural stability, feedback from visual system should be eliminated, which would enable capturing changes and understanding the characteristic of body posture instability in examined subjects [3, 6].

The examination of postural control can be based on performing targeted movement tasks by the patient, which is associated with performing the movement in a controlled and conscious way. Such tasks can be inadequate for the assessment of proprioceptive control, since only a minor part of proprioceptive stimuli become conscious [7]. The majority of information received by proprioceptors reaches mainly subcortical region of CNS (spinal cord, brainstem), which leads to transferring this information to subconsciousness [6]. The assessment of spine proprioception can be conducted with the use of passive movements with immobilization of parts of body or in nonupright positions [7, 43–46]. However, Preuss et al. stated that the examination of proprioceptive sensation is far more objective in upright positions than in nonweight-bearing positions and suggested that it may be the result of increased load of structures like intervertebral disc and of facet joints [42], which are one of the most frequent causes of LBP connected with mechanical overload of these structures.

### 4.2. Standardizing the Study Group for the Symptoms Can Influence the Reliability of Postural Control Assessment

Spine anomalies connected with mechanical damage like degenerative changes or herniated disc, visible on additional imaging tests (i.e., MRI and CT), are not always connected with the occurrence of pain, limited mobility of the spine or its functions [47, 48]. We believe that the diagnosis of patient with LBP should be based on clinical patterns [49], not on pathoanatomical changes, which in many cases are entirely asymptomatic [50, 51]. Such methodology of assessment enables to create homogeneous groups of LBP patients. It may lead to receiving more reliable and accurate results of the study and consequently improve the effectiveness of therapy of patients suffering from LBP.

Thus, it is crucial to standardize the study group for the symptoms using the MDT method and QTFC. The study group contained patients with LBP without radiation or with radiation only to proximal part of the lower limb according to QTFC, without neurological symptoms such as weakened muscle strength, asymmetry of reflexes, or weakened feeling within the nerve specific dermatome [20]. The examination included patients with LDS with the directional preference and centralization of symptoms [52]. Centralization and directional preference should be considered independent variables during the analysis of symptoms in the patient. Using the MDT method can prove either stricter inclusion, creating more homogeneous groups, or stricter experimental control in the smaller studies contributing to higher sensitivity of the research on postural control.

Changes in motor control in patients with LBP can cause further damage of this part of the spine, increasing the symptoms leading to fixation of faulty movement patterns [53, 54]. The decrease of afferent nerve fibers transferring the information from proprioceptors causes disrupted neuromuscular control and faulty static and dynamic postural control of the body through abnormal muscle activation patterns [55]. Thus, the postural control examination and proprioceptive training in patients with LBP should be inscribed in the algorithm of the examination and physiotherapeutic procedure.

### 4.3. Limitations of the Study

We are of the opinion that our study should include more patients with moderate and severe pain in the LBP group. What is more, although the study and the control group involved subjects between 18 and 35 year olds, they differed significantly from each other as regards age and body weight; nevertheless, they did not present weighty difference as far as BMI is concerned. BMI value in both groups was below 30, which may reduce the risk of LBP.

## 5. Conclusions

Summarizing, patients with LDS demonstrated significantly worse postural control in comparison with the healthy volunteers. This difference was visible while trials without visual control. Thus, it is vital to include the examination of body balance in the diagnostic algorithm in patients with LBP, as well as relevant proprioceptive training in these patients when necessary. We believe that it is crucial to standardize the procedure of postural control assessment in patients with LBP. Moreover, diagnostic procedures based on pain patterns (i.e., MDT) and the topography of pain (i.e., QTFC) seem equally important and can also contribute to receiving more reliable results of the study on postural control in this group of subjects.

## Figures and Tables

**Table 1:** Inclusion and exclusion criteria for the study and control groups.

Inclusion criteria for the study group	Inclusion criteria for the control group	Exclusion criteria for both groups
(i) Reporting pain of L-S parts areas of the spine for the minimum of 12 weeks (ii) At least one medical opinion and/or imaging test (i.e., MRI and CT) excluding cancer, spondylolisthesis, and congenital spinal malformations (iii) The degree of disability score ≥ 1% according to ODI questionnaire (iv) LDS—occurrence of centralisation and directional preference—according to MDT method (v) LBP without radiation or with radiation to the proximal part of lower limb—according to QTFC	(i) No LBP episodes inthe medical report	(i) Spine trauma (i.e., fracture) in medical report (ii) Surgical treatment in the area of spine and/or pelvis, lower limbs (iii) Neurologic diseases in medical report (np. MS, ALS) (iv) Rheumatoid diseases in the medical report (i.e., RA and AS) (v) DM (diabetic neuropathy) (vi) Blurred vision (vii) Pain in cervical spine on the day of the examination (viii) Vertigo symptoms in the medical report (ix) Pregnancy (x) Lack of cooperation (xi) Use of pain medications on the day of examination (xii) BMI ≥ 30

ODI: Oswestry Disability Index; MRI: magnetic resonance imaging; CT: computed tomography; LBP: low back pain; MS: multiple sclerosis; ALS: amyotrophic lateral sclerosis; RA: rheumatoid arthritis; AS: ankylosing spondylitis; DM: diabetes mellitus; BMI: body mass index, L-S: lumbosacral; LDS: lumbar derangement syndrome; QTFC: Quebec Task Force Classification.

**Table 2:** Subject characteristics.

No. of subjects	Study group			Control group			*p*
	Women	Men	All subjects	Women	Men	All subjects	
	37	33	70	36	20	50	
Variables	Mean	Mean	Mean	Mean	Mean	Mean	
Age (years)	27.54 ± 4.46	29.27 ± 3.72	28.35 ± 4.19	25 ± 3.59	25.45 ± 5.53	25.16 ± 4.34	<0.05
Height (cm)	169.21 ± 5.54	181.48 ± 7.89	175 ± 9.1	168.33 ± 6.19	181.3 ± 7.59	172.96 ± 9.14	>0.05
Body weight (kg)	62.29 ± 8.5	82.42 ± 8.64	71.78 ± 13.22	62.26 ± 7.23	75.15 ± 11.59	66.86 ± 10.88	<0.05
BMI (kg/m^2^)	21.73 ± 2.65	24.82 ± 2.44	23.19 ± 2.97	22 ± 2.58	22.84 ± 2.77	22.3 ± 2.66	>0.05
VAS (0-10)	2.18 ± 2.04	2.24 ± 2.22	2.21 ± 2.11	0	0	0	N/A
LBP (years)	4.57 ± 2.38	4.7 ± 2.39	4.63 ± 2.36	0	0	0	N/A

Values are mean ± SD. SD: standard deviation.

**Table 3:** Group characteristic according to the degree of disability (ODI) among study group participants.

Variables	Study group		
	Minimal disability (1%-20% ODI)	Moderate disability (21%-40% ODI)	Severe disability (41%-80% ODI)
No. of subjects (%)	37 (53%)	31 (44%)	2 (3%)
Age mean ± SD (y)	28.64 ± 4.36	27.93 ± 4.15	29.5 ± 0.7

ODI: Oswestry Disability Index; values are mean ± SD; SD: standard deviation.

**Table 4:** Group characteristic according to the level of pain (VAS) among study group participants after MDT assessment.

Variables	Study group			
	No LBP (0 VAS)	Minimal pain (1-3 VAS)	Moderate pain (4-6 VAS)	Severe pain (7-10 VAS)
No. of subjects (%)	25 (36%)	25 (36%)	17 (24%)	3 (4%)
Age ± SD (y)	27.16 ± 4.45	28.88 ± 3.9	29.05 ± 4.23	30 ± 3.6

VAS: Visual Analogue Scale; MDT: mechanical diagnosis and therapy; values are mean ± SD; SD: standard deviation.

**Table 5:** Group characteristic according to the level of physical activity (IPAQ).

Level of physical activity (IPAQ)	Study group			Control group			All subjects	*p*
	Women	Men	All	Women	Men	All		
Low	12	9	21	16	4	20	41	>0.05
	57.1%	42.9%	51.2%	80.0%	20.0%	48.8%		
Moderate	16	10	26	12	5	17	43	
	61.5%	38.5%	60.5%	70.6%	29.4%	39.5%		
High	9	14	23	8	11	19	42	
	39.1%	60.9%	54.8%	42.1%	57.9%	45.2%		
All subjects	37	33	70	36	20	56	126	

IPAQ: International Physical Activity Questionnaire.

## Data Availability

The data used to support the findings of this study may be released upon application to the Department of Rehabilitation Medicine, Medical University of Gdansk, who can be contacted at Marzena Olszewska-Karaban: marzena.olszewska-karaban@gumed.edu.pl.
